# Low serum sodium levels at hospital admission: Outcomes among 2.3 million hospitalized patients

**DOI:** 10.1371/journal.pone.0194379

**Published:** 2018-03-22

**Authors:** Saleem Al Mawed, V. Shane Pankratz, Kelly Chong, Matthew Sandoval, Maria-Eleni Roumelioti, Mark Unruh

**Affiliations:** 1 Division of Nephrology, Department of Internal Medicine, University of New Mexico School of Medicine, Albuquerque, New Mexico, United States of America; 2 University of New Mexico Clinical and Translational Science Center (CTSC), Albuquerque, New Mexico, United States of America; University Medical Center Groningen, NETHERLANDS

## Abstract

**Background:**

Hyponatremia is the most common electrolyte disorder among hospitalized patients. Controversies still exist over the relationship between hyponatremia and outcomes of hospitalized patients.

**Methods:**

To analyze the association of low serum sodium levels at hospital admission with in-hospital mortality and patient disposition and to compare the distribution of the risk of death associated with hyponatremia across the lifespan of hospitalized patients, we conducted an observational study of 2.3 million patients using data extracted from the Cerner Health Facts database between 2000 and 2014. Logistic regression models were used in the analyses.

**Results:**

At hospital admission 14.4% of hospitalized patients had serum sodium levels [Na] <135 mEq/L. In adjusted multinomial logistic regression analysis, we found that the risk of in-hospital mortality significantly increases for [Na] levels < 135 or ≥143 to ≤145 mEq/L compared to the reference interval of 140 to <143 mEq/L (p<0.001). We observed similar trends for the relationship between [Na] levels and discharge to hospice or to a nursing facility. We demonstrated that younger age groups (18 to <45, 45 to <65) had a higher risk of in-hospital mortality compared to older age groups (65 to <75, ≥75) for [Na] levels <130 mEq/L or 143 to ≤145 mEq/L (p<0.001).

**Conclusions:**

Hyponatremia is common among hospitalized patients and is significantly associated with in-hospital mortality, discharge to hospice or to a nursing facility. The risk of death and other outcomes was more evident for [Na] <135 mEq/L. The mortality associated with low [Na] was significantly higher in younger versus older patients.

## Introduction

Hyponatremia is the most frequent electrolyte disorder among hospitalized patients [1, 2]. It is associated with poor outcomes such as increased mortality, prolonged length of hospital stay, and increased healthcare costs [3–11]. Despite the wide recognition of the conventional definition of hyponatremia (serum sodium levels [Na] <135 mEq/L), different cutoff points were used by the various groups who have investigated the outcomes of hyponatremia in hospitalized patients [3–9, 12], with some investigators arguing that the current definition of hyponatremia should be reevaluated [8]. Hence, there are large variations in the prevalence of hyponatremia that range from 5.5% to 38% [4, 6–9]. Few studies have demonstrated harm associated with mild hyponatremia (130-134mEq/L) [7] or even low normal range of conventional normonatremia (<138 mEq/L) [8, 9]. However, there are also conflicting reports regarding whether mortality continues to increase as hyponatremia worsens [8, 13, 14]. Although some studies have shown an increase in mortality in older patients (>65 years) with hyponatremia [15–17], others have not been able to confirm this association [18, 19]. Furthermore, most of the studies addressing the relationship between hyponatremia and selected outcomes of hospitalized patients were single-center studies and did not include a large diverse population of hospitalized patients. Lastly, little is known about the association of hyponatremia with discharge disposition—specifically, discharge to hospice and nursing facility.

To address this gap in our knowledge regarding the cutoff points of hyponatremia associated with poor outcomes of hospitalized patients and the distribution of the risk of death associated with hyponatremia across the lifespan of these patients, we analyzed data on patients hospitalized between January 2000 and November 2014 derived from the Cerner Health Facts database.

## Materials and methods

### Study design, data source and population selection

We conducted an observational study of patients hospitalized between January 2000 and November 2014 using the Cerner Health Facts database. The study was done using the Health Facts database which is a national database that includes de-identified electronic health records (EHR) information from over 600 hospitals and clinics in the United States. The study was reviewed and approved by the University of New Mexico Institutional Review Board (15–531). The IRB waived the requirement for informed consent. We included the index hospital admission defined by the first inpatient encounter of any patient during the study period who met the following inclusion criteria: 1) age ≥ 18 years, and 2) first [Na] drawn during 24 hours before admission. The second inclusion criterion was chosen to reduce the possibility that the [Na] was affected by any treatments or iatrogenic causes after hospital admission.

**Fig 1:**
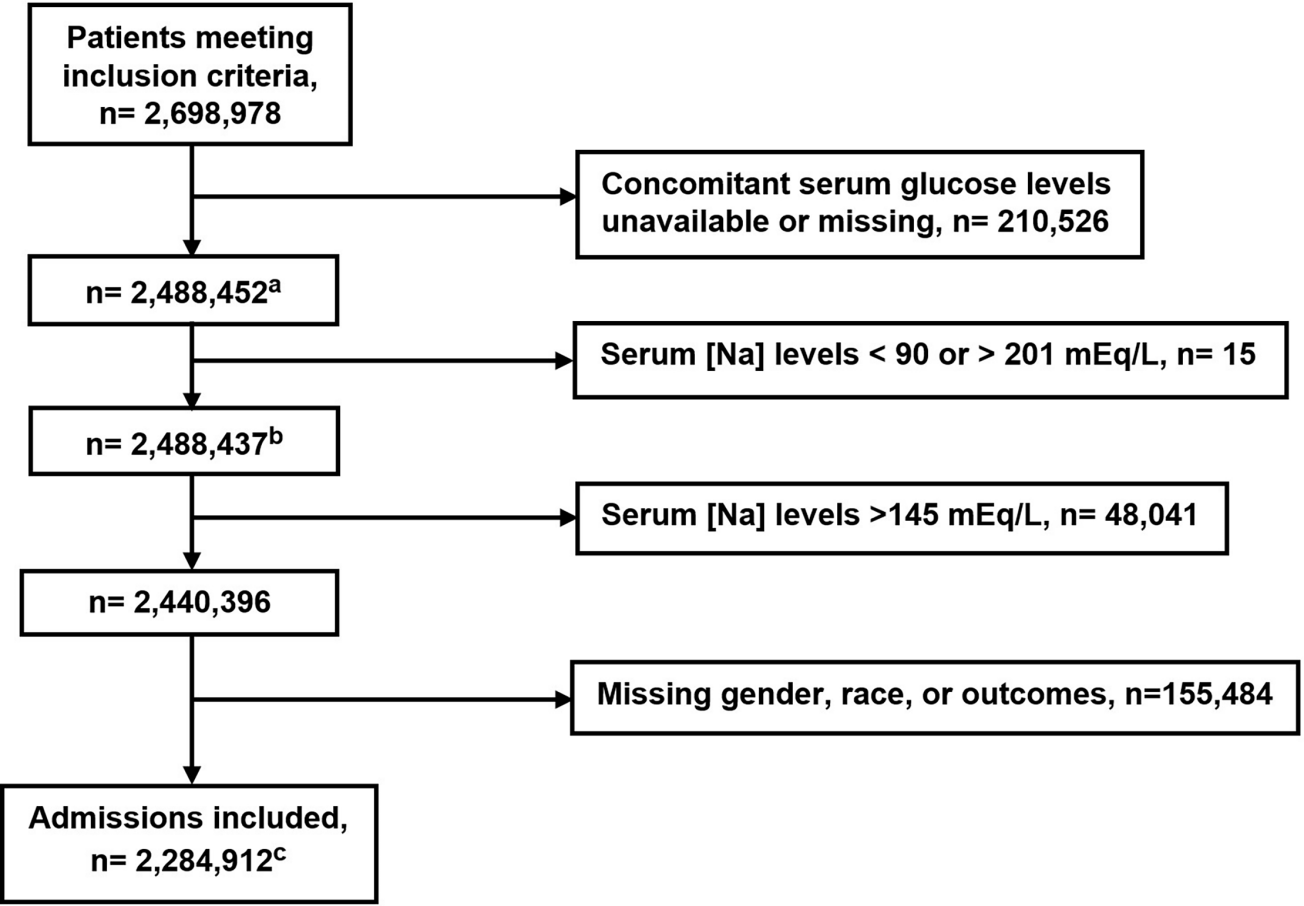
Flow chart of the sample selection process. ^**a**^Serum sodium levels were corrected by adding 1.6 mEq/L for each 100 mg/dL above 100 mg/dL of the concomitantly measured serum glucose levels. ^**b**^This number was used to calculate the prevalence of hyponatremia (Na < 135 mEq/L) at hospital admission. ^**c**^The final cohort available for analysis.

Information was gathered regarding patients’ demographics, co-morbidities, causes for admission, laboratory studies and disposition status at hospital discharge. The co-morbidities were identified using the International Classification of Diseases, 9th Edition, Clinical Modification (ICD-9-CM) codes, while the laboratory tests were identified using Logical Observation Identifiers Names and Codes (LOINC).The Deyo-Charlson Comorbidity Index (Deyo-CCI) was also calculated [20]. Deyo-CCI is a widely used co-morbidity index adapted from the Charlson co-morbidity index for administrative databases and uses ICD-9 codes. Since hyperglycemia is a hyperosmotic state affecting [Na], we corrected the [Na] by adding 1.6 mEq/L for each 100 mg/dL above 100 mg/dL of the concomitantly measured serum glucose levels [21]. After searching the literature for cases of extreme hyponatremia or extreme hypernatremia [22–25], we decided to exclude patients with [Na] <90 or >201 mEq/L. We further excluded patients with [Na] > 145 mEq/L and those with missing gender, race and outcomes values. The final cohort available for analysis included 2,284,912 patients (Fig 1). Our primary outcomes were in-hospital mortality (n = 63,359) and discharge disposition of hospitalized patients. The discharge dispositions included discharge to hospice (n = 32,335), discharge to home (n = 1,739,780), discharge to nursing facility (n = 274,755), discharge to short or long term care hospital (n = 91,703), rehabilitation (n = 58,259), and left against medical advice (n = 24,721). Discharge to hospice, discharge to nursing facility, and discharge to home were the main outcomes for discharge disposition.

### Statistical analyses

Continuous variables were summarized as means and standard deviations (SD) and were compared using student’s t tests. Categorical variables were summarized as percentages and were compared using chi-square tests.

We used logistic regression models to evaluate associations between patient characteristics and hyponatremia status (<135 mEq/L vs. ≥135 mEq/L). The patient characteristics of primary interest included age, gender, race and selected comorbidities and reasons for hospitalization (see Table 1). In these analyses, we categorized age into four different groups: 18 to <45, 45 to <65, 65 to <75, and ≥75 years old.

**Table 1:** Characteristics of hospitalized patients with or without hyponatremia.

Patient Characteristics*	All Cohort^a^ n = 2,488,437	Normonatremia and Hyponatremia patients^a^ (n = 2,440,396)	
		Presence of Hyponatremia (<135 mEq/L), n = 357,418	Absence of Hyponatremia (≥135 to ≤145 mEq/L), n = 2,082,978
Age, mean (SD), y	61.5 (19)	64.1 (18.1)	60.8 (19.1)
**Gender, %**			
Females	54.29	53.98	54.34
Males	45.69	46.01	45.64
N/A	0.02	0.01	0.02
**Race, %**			
Caucasians	74.46	78.28	73.97
African Americans	17.35	13.48	17.84
Hispanic	2.16	1.93	2.20
Native Americans	0.39	0.50	0.37
Pacific Islanders	0.06	0.07	0.06
Asians	0.98	1.05	0.96
Others	2.31	2.12	2.34
N/A	2.29	2.56	2.24
**Deyo-CCI, mean (SD)**	1.36 (1.89)	1.72 (2.25)	1.29 (1.81)
**Deyo-CCI categories, %**			
0	42.52	36.85	43.61
1	25.35	24.88	25.41
2	14.68	15.45	14.52
≥ 3	17.45	22.82	16.45
**Comorbidities, %**			
Hypertension	37.85	38.26	37.84
Myocardial Infarction	7.93	7.36	8.01
Coronary Artery Disease	20.82	19.74	21.05
Heart Failure	14.07	15.38	13.72
Diabetes Mellitus	21.96	24.97	21.40
Peripheral Vascular Disease	3.76	4.12	3.68
Chronic Kidney Disease	7.60	7.64	7.49
End Stage Renal Disease	2.42	3.34	2.28
Liver disease	2.44	4.73	2.04
Chronic Pulmonary disease	19.86	21.06	19.67
COPD	5.18	5.79	5.08
Adrenal Insufficiency	0.34	0.51	0.30
Hypothyroidism	9.32	10.52	9.12
Cerebrovascular disease	7.94	6.02	8.13
Hemiplegia/paraplegia	1.30	0.98	1.33
Rheumatologic disease	1.68	1.97	1.64
Depression	8.57	7.46	8.77
Dementia	3.5	2.57	3.42
AIDS	0.69	1.02	0.63
Malignancy^**b**^	7.84	11.39	7.26
Lung Cancer	2.07	3.85	1.77
**Reasons for Hospitalization, %**			
Pneumonia	4.81	6.20	4.55
Sepsis	2.46	3.58	2.16
SIADH	0.22	1.37	0.03
Urinary Tract Infection	6.62	7.91	6.23

N/A = not available; Deyo-CCI = Deyo Charlson Comorbidity Index; COPD = Chronic Obstructive Pulmonary Disease; AIDS = Acquired Immunodeficiency syndrome; SIADH = Syndrome of Inappropriate Antidiuretic Hormone.

* P<0.001 for the comparison between hyponatremic and normonatremic patients for all patient characteristics.

^**a**^Serum sodium levels corrected by adding 1.6 mEq/L for each 100 mg/dL above 100 mg/dL of the concomitantly measured serum glucose levels. These numbers are before excluding the patients with missing gender, race, or outcomes

^**b**^Except lung cancer

We were interested in evaluating broader associations between [Na] and outcomes. We therefore examined associations between quantitatively measured [Na] and our study outcomes in two ways. First, in order to visualize flexible relationships between [Na] and the outcomes, we modeled the associations using restricted cubic splines with five knots placed at 132, 136, 138, 140, and 143 mEq/L corresponding to the observed 5^th^, 27.5^th^, 50^th^, 72.5^th^, and 95^th^ percentiles of the cohort’s [Na] respectively. Second, for simplicity in interpretation, we formed the following eight [Na] categories: 1: < 120; 2: ≥ 120 to < 125; 3: ≥ 125 to < 130; 4: ≥ 130 to < 135; 5: ≥ 135 to < 138; 6: ≥ 138 to < 140; 7: ≥ 143 to ≤ 145 mEq/L and with 0: ≥ 140 to < 143 mEq/L as the reference group. We categorized the [Na] into 5 mEq/L intervals for [Na] between 120 and 135 mEq/L, as in previous studies. Since we had a very large number of patients with [Na] ≥ 135 mEq/L, we used narrower quantile-based intervals for higher [Na] to generate categories comprising of approximately equal numbers of patients in the higher categories.

As there were multiple interrelated and unordered outcomes, we used multinomial logistic regression models to assess associations between [Na] and the outcomes. These multinomial models allowed us to assess associations of each outcome while accounting for the competing risks of the others. In these models, we used “Discharge to home” as the reference outcome. We used the restricted cubic spline terms in these models to generate predicted probabilities across the continuum of [Na], and subsequently used these predictions in order to visualize the patterns of the associations between [Na] and the study outcomes. We used our defined [Na] categories in these models to examine associations between specific [Na] and the outcomes of interest, both with and without accounting for other covariates: age, gender, race, and the selected comorbidities and reasons for hospitalization outlined in Table 1.

We evaluated whether age has a differential effect on the relationships between [Na] and the outcomes. To accomplish this, we first used restricted cubic splines in age stratified multinomial logistic regression models to visualize trends in the predicted probabilities. We subsequently modeled the interaction between the age groups and the [Na] categories using multinomial logistic regression models while adjusting for age, gender, race, and the selected comorbidities and reasons for hospitalization. We also performed a sensitivity analysis after replacing the specific comorbidities with the Deyo-CCI in the covariate list.

In addition to examining associations between [Na] levels and the categories of primary outcomes, we also assessed the relationships between [Na] levels and length of hospitalization among those who were discharged to home. We used linear regression approaches to model the number of days of hospitalization with [Na] categories, while adjusting for age, gender, race, and the selected comorbidities and reasons for hospitalization. We additionally tested for the differential effect of age on these associations. In order to meet the model assumptions, we performed analyses after logarithmic transformation of the length of stay variable, and reported results after back-transformation to the original scale. All analyses were performed using Stata version 14.1 (StataCorp LP, College Station, TX).

## Results

We found 2,698,978 patients who met our inclusion criteria. From this cohort 210,526 patients were lacking a concomitant serum glucose determination and were excluded from the study cohort. Our initial cohort of patients included 2,488,437 patients (Fig 1). The prevalence of hyponatremia ([Na] <135 mEq/L) at hospital admission was 14.4% (357,418).

Patients with hyponatremia were older than patients with normonatremia ([Na] = 135–145 mEq/L), 64.1±18.1 vs. 60.8 ±19.1 years old (p<0.001). Patients with hyponatremia had a higher overall Deyo-CCI score compared with normonatremic patients 1.72±2.25 vs. 1.29±1.81, respectively, (p<0.001). Other pertinent patient characteristics are summarized in Table 1.

We found that hyponatremia increases with increasing age after controlling for gender, race and the selected comorbidities and reasons for hospitalization. The odds ratios (ORs) [95% confidence interval, CI] of having hyponatremia for ages 45 to <65, 65 to <75 and 75 were 1.21 [1.20–1.23], 1.35 [1.33–1.37] and 1.56 [1.54–1.58], respectively, compared to the reference age group 18 to <45 years old (S1 Table).

### Low serum sodium levels and the outcomes

**Fig 2:**
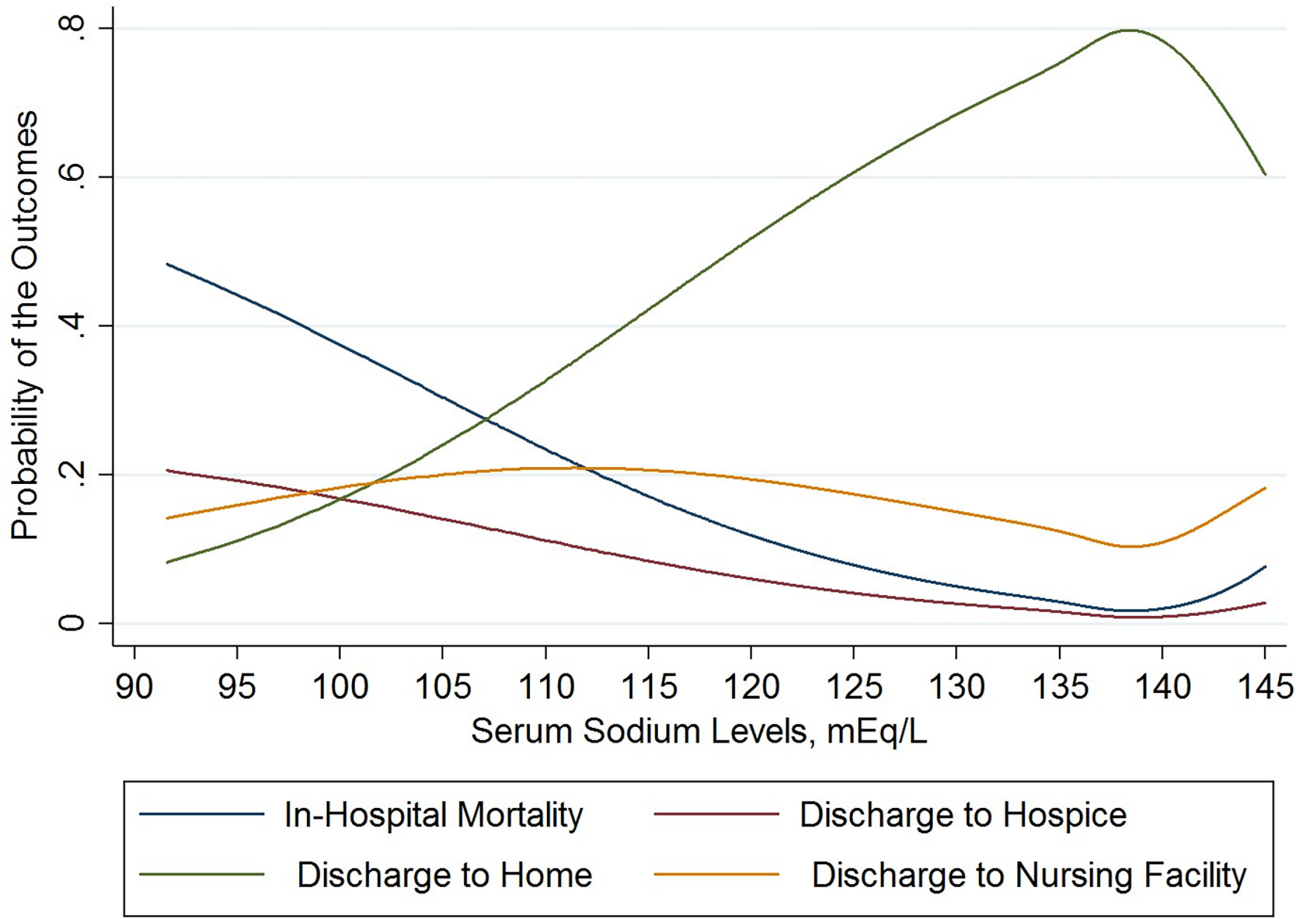
Restricted cubic splines of the crude probability of in-hospital mortality, discharge to hospice, discharge to home, and discharge to nursing facility as a function of serum sodium levels at hospital admission. These estimated probabilities were derived from a multinomial logistic regression model. Serum sodium levels were corrected by adding 1.6 mEq/L for each 100 mg/dL above 100 mg/dL of the concomitantly measured serum glucose levels.

The crude probability of in-hospital mortality corresponding to [Na] was lowest between 135 and 143 mEq/L and continued to increase above or below this range. The probability of discharge to hospice demonstrated a similar pattern, though less profound. The estimated probability of discharge to nursing facility also demonstrated a similar pattern but started to decrease at [Na] below 115mEq/L. We observed the opposite for discharge to home, such that the probability increased up to [Na] between 135 and 143 mEq/L, then decreased above this range (Fig 2).

For the [Na] categories, the crude in-hospital mortality increased as [Na] decreased or increased relative to the 138 to <140 mEq/L interval. This was also true for discharge to hospice and discharge to nursing facility. However, the percentage of discharge to nursing facility decreased for [Na] intervals 120 to <125 mEq/L and <120mEq/L (Table 2).

**Table 2:** Relationship between serum sodium levels at hospital admission and in-hospital mortality, discharge to hospice or to nursing facility.

Serum [Na] levels^a^ at Hospital Admission (mEq/L), n = 2,284,912		In-Hospital Mortality (n = 63,359)		Discharge to Hospice (n = 32,335)		Discharge to Nursing Facility (n = 274,755)	
		In-Hospital Mortality, (%)	Adjusted^b^ Relative Risk Ratio (95% CI)	Discharge to Hospice, n (%)	Adjusted^b^ Relative Risk Ratio (95% CI)	Discharge to Nursing Facility, n (%)	Adjusted^b^ Relative Risk Ratio (95% CI)
**Absence of Hyponatremia (≥135 to ≤145 mEq/L), n = 1,950,594**	**143 to ≤ 145** (n = 134,979)	7,242 (5.4%)^**c**^	**2.15 (2.08–2.22)**	2,981(2.2%)	**1.91 (1.82–2.00)**	22,085 (16.4%)	**1.36 (1.33–1.38)**
	**140 to < 143** (n = 602,058)	**15,112 (2.5%)**	**1 (reference)**	**7,082 (1.2%)**	**1 (reference)**	**71,770 (11.9%)**	**1 (reference)**
	**138 to < 140** (n = 601,610)	11,637 (1.9%)	**0.78 (0.76–0.80)**	5,997 (1%)	**0.86 (0.83–0.89)**	63,865 (10.6%)	**0.97 (0.96–0.99)**
	**135 to < 138** (n = 611,947)	13,953 (2.3%)	**0.90 (0.88–0.92)**	7,809 (1.3%)	**1.06 (1.03–1.10)**	68,836 (11.2%)	**1.08 (1.07–1.10)**
**Presence of Hyponatremia (<135 mEq/L), n = 334,318**	**130 to < 135** (n = 280,970)	11,475 (4.1%)	**1.45 (1.41–1.49)**	6,369 (2.3%)	**1.64 (1.58–1.70)**	39,416 (14.0%)	**1.26 (1.24–1.28)**
	**125 to < 130** (n = 41,953)	2,941 (7.0%)	**2.43 (2.32–2.54)**	1,579 (3.8%)	**2.56 (2.41–2.72)**	7,038 (16.8%)	**1.35 (1.31–1.39)**
	**120 to < 125** (n = 8,982)	739 (8.2%)	**3.12 (2.87–3.40)**	408 (4.5%)	**3.25 (2.91–3.63)**	1,385 (15.4%)	**1.21 (1.14–1.29)**
	**< 120** (n = 2,413)	260 (10.8%)	**4.85 (4.20–5.60)**	110 (4.6%)	**3.71 (3.01–4.56)**	360 (14.9%)	**1.32 (1.16–1.49)**

CI = confidence interval.

^**a**^Serum sodium levels corrected by adding 1.6 mEq/L for each 100 mg/dL above 100 mg/dL of the concomitantly measured serum glucose levels.

^**b**^The adjusted relative risk ratios were derived from a multinomial logistic regression model adjusted for age, gender, race, and the selected comorbidities and reasons for hospitalization (p<0.001 for all). Discharge to home is the referent outcome (n = 1,739,780).

^**c**^The percentages represent the percentage of each outcome within each serum sodium category.

**Fig 3:**
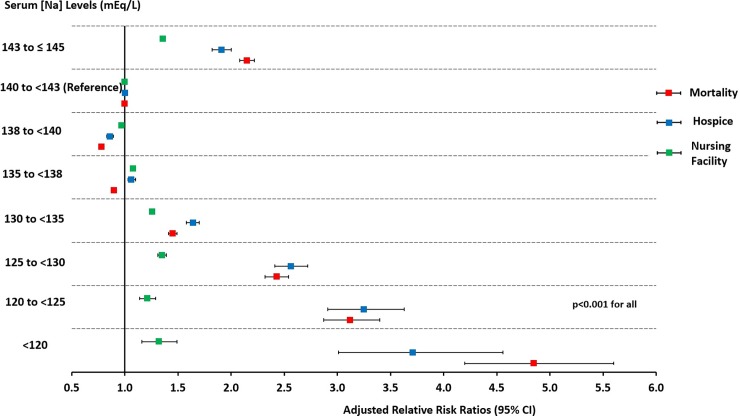
Forest plot of the relative risk ratios (95% CI) for in-hospital mortality, discharge to hospice, or discharge to nursing facility associated with different intervals of serum sodium levels (mEq/L) at hospital admission. The relative risk rations were derived from multinomial logistic regression models adjusted for age, gender, race, and the selected comorbidities and reasons for hospitalization. Discharge to home and serum sodium levels of (140 to <143 mEq/L) served as referent. Serum sodium levels were corrected by adding 1.6 mEq/L for each 100 mg/dL increase above 100 mg/dL of the concomitantly measured serum glucose levels. Error bars indicated 95% CI. CI = confidence interval.

In the adjusted multinomial logistic regression analysis, we found that the likelihood of in-hospital mortality significantly increased for [Na] < 135 mEq/L or ≥ 143 to ≤145 mEq/L compared to the reference interval of 140 to <143 mEq/L (p<0.001). Patients with [Na] of <120, 120 to <125, 125 to <130, 130 to <135, 135 to <138, 138 to <140 and 143 to ≤145 mEq/L compared with 140 to<143 mEq/L had in-hospital mortality relative risk ratios [95% CI] of 4.85 [4.20–5.60], 3.12 [2.87–3.40], 2.43 [2.32–2.54], 1.45 [1.41–1.49], 0.90 [0.88–0.92], 0.78 [0.76–0.80] and 2.15 [2.08–2.22] respectively (p<0.001 for all) (Table 2 and Fig 3).

This strong association with in-hospital mortality continued to rise with the increase in the severity of hyponatremia (Table 2 and Fig 3). Patients with [Na] of 138 to < 140 mEq/L had the lowest likelihood of in-hospital mortality. We observed similar trends for the relationship between [Na] and discharge to hospice or to a nursing facility (Table 2 and Fig 3). We obtained very similar results after adjusting for the Deyo-CCI instead of the selected comorbidities (S2 Table and S1 Fig).

There was a significant association between [Na] categories and length of hospitalization (p<0.001). These overall relationships are shown in (S3 Table). For those discharged to their homes, length of stay [95% CI] was 16% [14%-19%] to 29% [24%-35%] longer when [Na] levels were below 135 mEq/L. Even when [Na] levels were above 135 mEq/L, there was significant variability in length of stay (p<0.001), although the relative differences were smaller. Length of stay was shortest when [Na] levels were between 140 and <143 mEq/L. Lengths of stay in the normonatremia range were longest when they were on the lowest end of the scale, with stays being 12% longer for those with [Na] between 135 and <138 mEq/L. However, even those with [Na] levels on the highest end of the normal [Na] range (143–145 mEq/L) experienced a slightly longer length of stay 3% [2% - 3%] longer than those in the [Na] category between 140 and <143 mEg/L.

### Age and the outcomes

**Fig 4:**
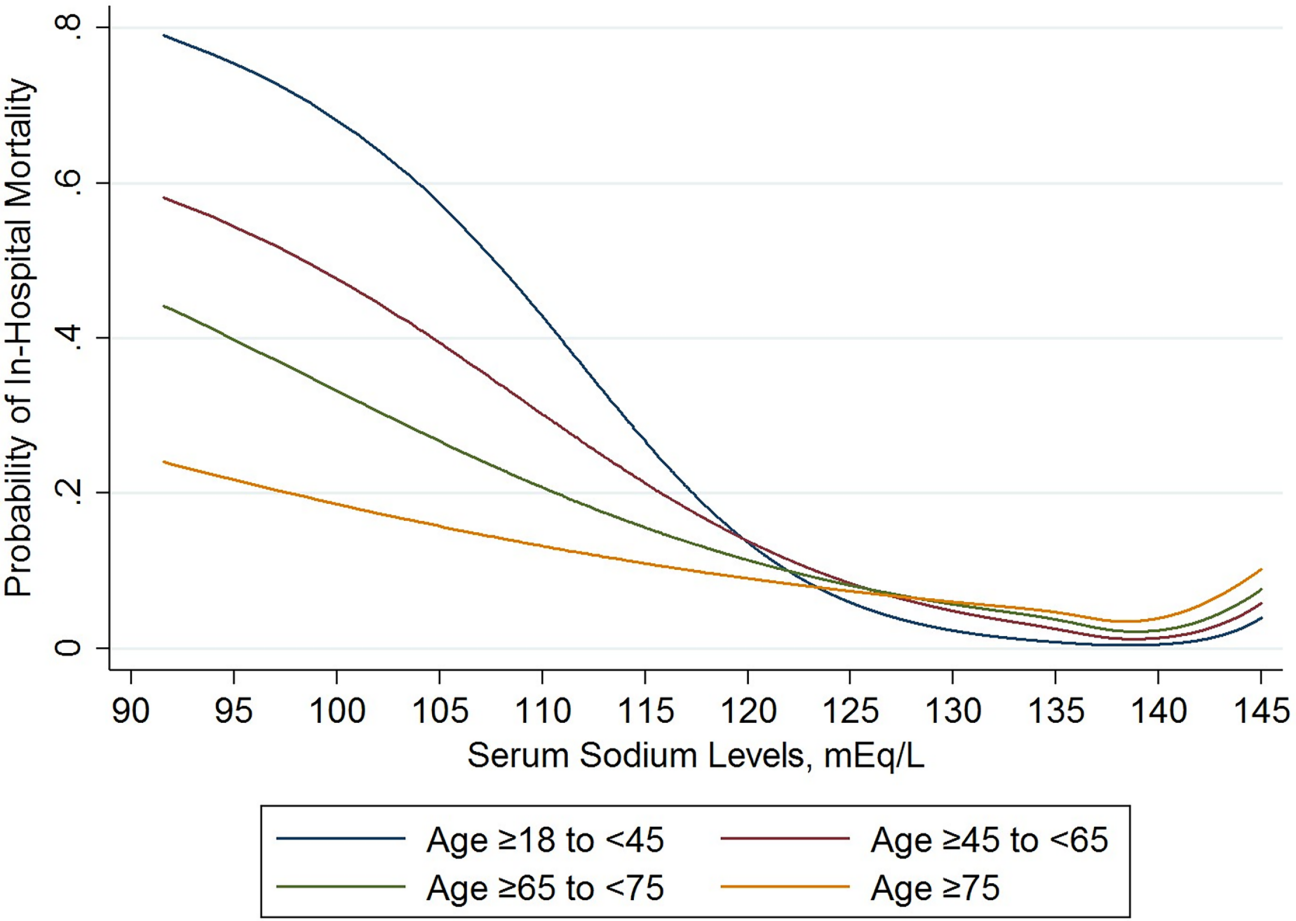
Restricted cubic splines of the estimated probability of in-hospital mortality as a function of serum sodium levels for the different age groups. These estimated probabilities were derived from a multinomial logistic regression models stratified by age.

We observed that for low [Na], the probability of in-hospital mortality was higher for younger age groups compared to older age groups (Fig 4). For discharge to hospice, as [Na] decreased the probability for the youngest (18 to <45) and oldest (≥75) age groups, was lower than that for the middle age groups (45 to <65 and 65 to <75) (S2 Fig). For discharge to nursing facility, older age groups had higher probabilities compared to younger age groups for all [Na] (S3 Fig). For normal [Na], the probability for discharge to home was higher in the younger age groups compared to older age groups. However, as the [Na] decreased, we observed the opposite (S4 Fig).

**Fig 5:**
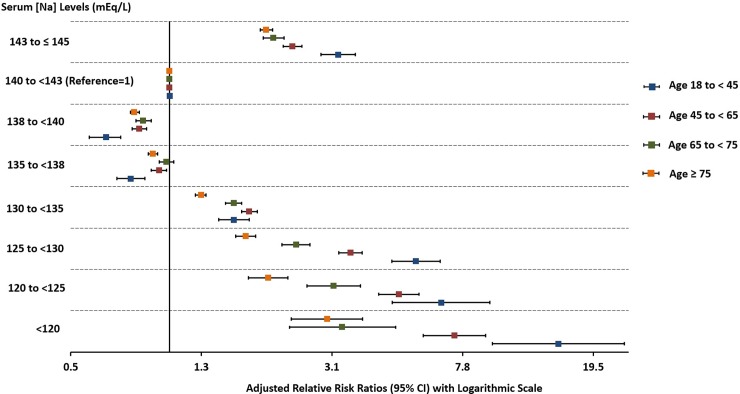
Forest plot of the relative risk ratios (95% CI) for in-hospital mortality associated with different intervals of serum sodium levels (mEq/L) at hospital admission for the different age groups. The relative risk ratios were derived from multinomial logistic regression models adjusted for age, gender, race, and the selected comorbidities and reasons for hospitalization. Serum sodium levels of (140 to <143 mEq/L) served as referent and each age group served as its own referent. Serum sodium levels were corrected by adding 1.6 mEq/L for each 100 mg/dL increase above 100 mg/dL of the concomitantly measured serum glucose levels. Error bars indicated 95% CI. CI = confidence interval. p<0.001 for all except for age 65 to <75 in the 135 to <138 mEq/L category (p = 0.43).

We obtained similar findings after modeling the interaction between each age group and the [Na] categories (Fig 5 and S4 Table). The global test of interaction between age and [Na] categories demonstrated a significant interaction between these two factors and in-hospital mortality (p<0.001), and almost all individual interaction terms were similarly significant, with the exception of age 65 to <75 in the 135 to <138 mEq/L category when compared to the same age group in the 140 to <143 mEq/L category (p = 0.43) (Fig 5 and S4 Table). We compared the relative risk ratios for in-hospital mortality associated with specific [Na] categories between the different age groupings from the model that included the age by [Na] interaction (S5 Table). We noted that for [Na] <130 mEq/L and ≥143 to ≤145 mEq/L, the relative risk ratios for in-hospital mortality were significantly greater for each age group compared to the consecutive older age group, except for ages 65 to <75 and ≥ 75 in the [Na] category of <120 mEq/L, 18 to <45 and 45 to <65 in the [Na] category of 120 to <125 mEq/L, and 65 to <75 and ≥ 75 in the [Na] category of 143 to ≤ 143 mEq/L. However, for ≥135 to <138 and ≥138 to <140 mEq/L [Na] categories, the relative risk ratios for in-hospital mortality were significantly lower in the 18 to < 45 age groups compared to all other older age groups (S5 Table).

There were also significant age differences in associations between hospitalization length and [Na] categories (p<0.001) for patients who were discharged to their homes (S5 Fig). Patients between 18 and <45 years of age had the shortest lengths of stay for [Na] categories less than 135 mEq/L, but they experienced among the longest lengths of stay for very low [Na] levels. Patients of at least 75 years of age experienced the least variation in length of stay as a function of [Na] levels. The associations between [Na] levels and length of stay, by age, were found to be consistent with the associations observed for the other outcomes.

## Discussion

In this observational study using a large multicenter database, we were able to demonstrate using the cutoff point of <135 mEq/L that the prevalence of hyponatremia was common at hospital admission (14.4%). We showed that having [Na] < 135 mEq/L or ≥ 143 to ≤145 mEq/L is a significant risk factor for in-hospital mortality and increased discharge to hospice or to a nursing facility. Moreover, we found that younger patients admitted with [Na] <130 mEq/L or ≥143 to ≤ 145 mEq/L had a higher risk of in-hospital mortality compared to older patients.

In general, our results corroborate those of several other investigators. For example, Waikar et al [7] reported that the prevalence of hyponatremia at hospital admission in two teaching hospitals with 98,411 patients was 14.5% and in another study by Holland-Bill et al [14], which included 279,508 hospitalized patients, using the same cutoff point of <135 mEq/L the prevalence was 15%. In another study by Wald et al, which included 53,236 adult hospitalizations, the prevalence of hyponatremia at hospital admission was higher (38%) [8]. However, using the same cutoff point used by Wald et al (<138 mEq/L) yielded us a very similar prevalence (40.56%).

Our results are in accordance to previous studies showing that hyponatremia is an independent risk factor for in-hospital mortality [7, 8, 14]. Different mechanisms have been proposed to explain the association between hyponatremia and mortality [26, 27]. These mechanisms attempt to describe whether hyponatremia is a direct cause of mortality or whether hyponatremia is only a complication of other severe medical conditions which themselves lead to death. The first possibility can be explained by the direct effect of acute hyponatremia on severe brain edema, encephalopathy, and brain herniation, which subsequently lead to death. The second possibility can arise from multiple situations where hyponatremia is caused by other serious medical conditions such as advanced heart failure or cirrhosis. There is a third possibility as well: hyponatremia may have an additive effect on mortality risk in the presence of other serious medical conditions. Comorbid conditions can cause increased mortality alone and can also lead to further increased mortality risk through subsequent development of hyponatremia. Hyponatremia on the other hand, can further increase the risk of mortality independent of the underlying disease, as has been suggested by previous studies which have found an independent association between hyponatremia and the risk of in-hospital mortality even after adjusting for the potential confounding factors [26, 27].

We documented a significant association between hyponatremia and the increased risk of discharge to hospice or to a nursing facility. Furthermore, we demonstrated that the in-hospital mortality continues to worsen with the increase in the severity of hyponatremia, a conclusion that was also reached by Wald et al [8]. However, this conclusion is at odds with the findings of Holland-Bill et al [14]. In that study the risk of mortality increased as the [Na] decreased; however, when [Na] values dropped to <132 mEq/L there was no further increase in mortality. Also, our results are in disagreement with the findings of the study by Chawla et al that included 45,693 hospitalized patients from a single hospital between 1996 and 2007. In this study, the in-hospital mortality increased as [Na] decreased from 134 to 120 mEq/L [13]. However, there was an opposite pattern for [Na] below 120 mEq/L where the mortality rate decreased progressively.

Although in the present study the risk of in-hospital mortality was the lowest in the 138 to <140 mEq/L group, the in-hospital mortality for the 135 to < 138 mEq/L group was still low compared to the other [Na] categories. Our results support the traditional lower cutoff point of normonatremia of 135 mEq/L. Realizing that the risk of in-hospital mortality intensified for values ≥ 143 to ≤145 mEq/L, we support the proposition expressed by Wald et al that the traditional higher cutoff point of normonatremia (≤ 145 mEq/L) needs to be reevaluated.

We demonstrated that the likelihood of in-hospital mortality associated with [Na] levels varied between the various age groups. Younger age groups (18 to <45 years and 45 to <65 years) had higher likelihood of in-hospital mortality compared to older age groups (65 to <75 years, ≥75 years) for [Na] <130 mEq/L or ≥143 to ≤ 145 mEq/L. It is the contention of some investigators that elderly patients have a high risk of mortality at hospital admission [15–17]. However, other investigators do not agree with this position [18, 19]. In a retrospective study by Ganguli and colleagues that included 608 elderly participants aged >65 years in a home-based primary care program, it was found that hyponatremia was independently associated with falls, fractures, hospitalizations but not mortality [19]. In a case control study by Ahamed et al, including patients aged ≥ 65 years old admitted to the hospital during a 6 months period, hyponatremia was independently associated with admission-associated falls and increased length of hospital stay but was not associated with increased risk of mortality [18]. Moreover, Wald et al [8] compared the mortality rates of patients younger than 65 years old vs. patients who were at least 65 years old and found that the in-hospital mortality rate was higher in younger patients compared to older patients.

The differential association between hyponatremia and mortality in the different age groups has several plausible explanations. It is well known that chronic hyponatremia (>48 hours) is very common in elderly population: chronic hyponatremia is present in 11.3% of elderly people living in the community [28] and 18% of the elderly residing in nursing home facilities [29]. In chronic hyponatremia, adaptation mechanisms in the brain render patients less prone to the development of brain herniation. Moreover, the possibility of developing osmotic demyelination after the correction of chronic hyponatremia is rare and less likely if the hyponatremia is corrected slowly [30]. The other possible explanation is the difference in the ratio of the brain size to the cranial vault between elderly patients and younger patients. The high ratio of the size of the brain to the cranial vault in children has been postulated to play and important role in impeding brain adaptation mechanisms and leading to the observed increased mortality for those with hyponatremia [31]. Hence, it is believed that the decrease in brain volume due to brain atrophy in the elderly plays a protective role against the sequelae of hyponatremic encephalopathy [32]. In contrast, younger patients probably suffer from acute hyponatremia which develops over the course of few hours and most probably die from cerebral edema and brain herniation [30]. Due to the inhibitory effects of estrogen on the brain adaptation to hyponatremia, menstruating women have 25 times higher risk of death or permanent neurological damage from hyponatremic encephalopathy than postmenopausal women [32]. Furthermore, younger patients are more likely to engage in risky behaviors such as ecstasy use [33] and marathon running [34] that are linked to fatal complications of hyponatremia.

Our study has several strengths. The size of our study allowed us to robustly investigate the association between the different levels of hyponatremia and selected outcomes of hospitalized patients even with very low levels of hyponatremia. For example, for patients with serum sodium levels < 120 mEq/L the number of deaths was 260 out of 2413, which, to our knowledge, is higher than that found in any other published report. Our cohort included a diverse patient population from many hospitals in the United States, hence our results are generalizable to the US population.

Our study also had several limitations. First, inherent in our study was the use of ICD-9 codes to identify patients’ comorbidities, which is prone to billing and coding errors. However, the Health Facts data undergo extensive review before being added to the database. Second, the identification of the comorbidities and reason for hospitalization using ICD-9 codes in our study does not account for the severity of these medical conditions. Due to the limitation of our observational study, we were not able to include a disease severity scoring modality such as the Acute Physiology and Chronic Health Evaluation (APACHE) or Sequential Organ Failure Assessment (SOFA). This may have led to unmeasured confounding. However, we were able to adjust for the Deyo-Charlson Comorbidity Index (Deyo-CCI) which has been used previously by studies that demonstrated an independent association between hyponatremia and in-hospital mortality [7, 8, 14]. The Deyo-CCI has been widely used to predict survival of hospitalized patients and shown to have good utility in predicting in-hospital mortality in critically ill patients [35]. Third, we based our interpretation of the relationship between in-hospital mortality due to hyponatremia and age on the assumption that chronic hyponatremia is common in older patients and acute hyponatremia is common in younger patients. We did not have the [Na] data in the outpatient setting which is very important to distinguish chronic from acute hyponatremia. Fourth, we also did not have the information about the use of the outpatient medications that predispose to the development of hyponatremia. Including the data about these medications would enhance the information provided in our study. Moreover, confirming that the elderly patients in our study are in fact taking the medications that are known to cause chronic hyponatremia will make us more comfortable in stating our assumption that chronic hyponatremia was more common in these patients. Nevertheless, we believe that accounting for the real causes of hyponatremia or determining the exact mechanism for the association between hyponatremia and in-hospital mortality should not distract us from the important finding in our study that hyponatremia, regardless of the cause, is significantly associated with increased in-hospital mortality. Finally, because of the observational nature of our study we cannot preclude the possibility of residual confounding and cannot draw any causal interpretations from our results. However, conducting large randomized controlled trails (RCT) to overcome this limitation may not be feasible.

## Conclusions

Hyponatremia is common among hospitalized patients and regardless of the cause, it contributes significantly to the morbidity and mortality of hospitalized patients. It is an important risk factor for increased in-hospital mortality and increased discharge to hospice or to a nursing facility. Moreover, younger patients admitted with hyponatremia had a higher risk of in-hospital mortality compared to older patients. Our study underscores the need for more alertness to the [Na] in the outpatient setting and emphasizes the need for providing equal attention and care to all patients presenting to hospital with hyponatremia. Studies are needed to evaluate the traditional higher cutoff point of normonatremia (≤ 145 mEq/L).

## Supporting information

S1 Table Factors associated with hyponatremia (<135 mEq/L). OR = Odds Ratio; Ref = Reference; DM = Diabetes Milletus; ESRD = End Stage Renal Disease; AIDS = Acquired Immunodeficiency syndrome; SIADH = Syndrome of Inappropriate Antidiuretic Hormone; UTI = Urinary Tract Infection.
*Adjusted for age, gender, race and selected comorbidities and reasons for hospitalization.
(DOCX)

S2 Table Relationship between serum sodium levels at hospital admission and in-hospital mortality, discharge to hospice or to nursing facility (Adjusting for the Deyo-CCI). CI = confidence interval; Deyo-CCI = Deyo-Charlson Comorbidity Index.
^**a**^ Serum Sodium Levels corrected by adding 1.6 mEq/L for each 100 mg/dL above 100 mg/dL of the concomitantly measured serum glucose levels.
^**b**^The adjusted relative risk ratios were derived from a multinomial logistic regression model adjusted for age, gender, race, and the Deyo-CCI (p<0.001 for all).
(DOCX)

S3 Table Relationships between serum sodium levels at hospital admission and length of hospitalization among those discharged to home. CI = confidence interval.
^**a**^Serum sodium levels corrected by adding 1.6 mEq/L for each 100 mg/dL above 100 mg/dL of the concomitantly measured serum glucose levels.
^**b**^The adjusted relative ratios were derived from linear regression model adjusted for age, gender, race, and the selected comorbidities and reasons for hospitalization (p<0.001 for all).
(DOCX)

S4 Table In-hospital mortality associated with different intervals of serum sodium levels for the different age groups. ^**a**^The percentages and the relative risk ratios (RRR) of in-hospital mortality are for each age group with the respective [Na] category.
^**b**^The relative risk ratios are derived from a multinomial logistic regression model adjusted for age, gender, race, and the selected comorbidities and reasons for hospitalization. CI = Confidence interval. p<0.001 for all except: ^**c**^ p = 0.45.
(DOCX)

S5 Table Comparisons of the relative risk ratios of in-hospital mortality associated with the different serum sodium levels intervals for the different age groups. RRR = Relative Risk Ratio.
Age0 = Age (≥18 to <45); Age1 = Age (≥45 to <65); Age2 = Age (≥65 to <75); Age3 = Age ≥75.
(DOCX)

S1 Fig Forest plot of the relative risk ratios (95% CI) for in-hospital mortality, discharge to hospice, or discharge to nursing facility associated with different intervals of serum sodium levels (mEq/L) at hospital admission. The relative risk ratios were derived from multinomial logistic regression models adjusted for age, gender, race, and Deyo-CCI. Discharge to home and serum sodium levels of (140 to <143 mEq/L) served as referent. Serum sodium levels were corrected by adding 1.6 mEq/L for each 100 mg/dL increase above 100 mg/dL of the concomitantly measured serum glucose levels. Error bars indicated 95% CI. CI = confidence interval; Deyo-CCI = Deyo Charlson Comorbidity Index.
(DOCX)

S2 Fig Restricted cubic splines of the estimated probability of discharge to hospice as a function of serum sodium levels for the different age groups. These estimated probabilities were derived from a multinomial logistic regression models stratified by age.
(DOCX)

S3 Fig Restricted cubic splines of the estimated probability of discharge to nursing facility as a function of serum sodium levels for the different age groups. These estimated probabilities were derived from a multinomial logistic regression models stratified by age.
(DOCX)

S4 Fig Restricted cubic splines of the estimated probability of discharge to home as a function of serum sodium levels for the different age groups. These estimated probabilities were derived from a multinomial logistic regression models stratified by age.
(DOCX)

S5 Fig Forest plot of the estimated lengths of hospitalization in days (and 95% CI) associated with different intervals of serum sodium levels (mEq/L) at hospital admission for individuals in different age categories. These estimated geometric means were derived from a linear regression model on the logarithmic-transformed number of days of hospitalization on [Na] levels with age categories included both as main effects, and in interactions with [Na] levels, while also adjusting for age, gender, race, and Deyo-CCI.
(DOCX)
