# Supplementary material for: Low serum sodium levels at hospital admission: Outcomes among 2.3 million hospitalized patients
Source: PLoS One. 2018 Mar 22;13(3):e0194379. doi: 10.1371/journal.pone.0194379 (PMC5864034; doi:10.1371/journal.pone.0194379)
Supplement: S1 Table — OR = Odds Ratio; Ref = Reference; DM = Diabetes Milletus; ESRD = End Stage Renal Disease; AIDS = Acquired Immunodeficiency syndrome; SIADH = Syndrome of Inappropriate Antidiuretic Hormone; UTI = Urinary Tract Infection. *Adjusted for age, gender, race and selected comorbidities and reasons for hospitalization. (DOCX) [file pone.0194379.s001.docx]

|  | **Multivariate Logistic Regression** | |
| --- | --- | --- |
| **Variables Included in the Model** | **Adjusted^*^ OR (95% CI)** | **P value** |
| **Age** (Ref: Age 18 to <45) |  |  |
| **45 to <65**  **65 to <75**  **≥75** | 1.21 (1.20-1.23)  1.35 (1.33-1.37)  1.56 (1.54-1.58) | <0.001  <0.001  <0.001 |
| **Comorbidities** | | |
| **DM** (Ref: No DM) | 1.25 (1.24-1.26) | <0.001 |
| **ESRD** (Ref: No ESRD) | 1.50 (1.47-1.53) | <0.001 |
| **Chronic Liver Disease** (Ref: No Chronic Liver Disease | 1.34 (1.28-1.39) | <0.001 |
| **Cirrhosis** (Ref: No Cirrhosis) | 2.67 (2.61-2.73) | <0.001 |
| **Adrenal Insufficiency** (Ref: No Adrenal Insufficiency) | 1.44 (1.37-1.52) | <0.001 |
| **AIDS** (Ref: No AIDS) | 1.98 (1.90-2.05) | <0.001 |
| **Lung Cancer** (Ref: No Lung Cancer) | 1.47 (1.44-1.51) | <0.001 |
| **Pneumonia** (Ref: No Pneumonia) | 1.33 (1.31-1.35) | <0.001 |
| **Sepsis** (Ref: No Sepsis) | 1.59 (1.57-1.62) | <0.001 |
| **SIADH** (Ref: No SIADH) | 40.91 (37.68-44.41) | <0.001 |
| **UTI** (Ref: No UTI) | 1.30 (1.29-1.31) | <0.001 |
