# Supplementary material for: Low serum sodium levels at hospital admission: Outcomes among 2.3 million hospitalized patients
Source: PLoS One. 2018 Mar 22;13(3):e0194379. doi: 10.1371/journal.pone.0194379 (PMC5864034; doi:10.1371/journal.pone.0194379)
Supplement: S2 Table — CI = confidence interval; Deyo-CCI = Deyo-Charlson Comorbidity Index. a Serum Sodium Levels corrected by adding 1.6 mEq/L for each 100 mg/dL above 100 mg/dL of the concomitantly measured serum glucose levels. bThe adjusted relative risk ratios were derived from a multinomial logistic regression model adjusted for age, gender, race, and the Deyo-CCI (p<0.001 for all). (DOCX) [file pone.0194379.s002.docx]

| **Serum [Na] levels^a^ at Hospital Admission (mEq/L), n= 2,284,912** | | **In-Hospital Mortality (n=63,359)** | **Discharge to Hospice (n=32,335)** | **Discharge to Nursing Facility (n=274,755)** |
| --- | --- | --- | --- | --- |
|  |  | **Adjusted^b^ Relative Risk Ratio (95% CI)** | **Adjusted^b^ Relative Risk Ratio (95% CI)** | **Adjusted^b^ Relative Risk Ratio (95% CI)** |
| **Absence of Hyponatremia (≥135 to ≤145 mEq/L), n=1,950,594** | **143 to ≤ 145** (n=134,979) | **2.30 (2.23-2.36)** | **2.00 (1.91-2.09)** | **1.43 (1.40-1.46)** |
|  | **140 to < 143** (n=602,058) | **1 (reference)** | **1 (reference)** | **1 (reference)** |
|  | **138 to < 140** (n=601,610) | **0.78 (0.76-0.79)** | **0.85 (0.82-0.88)** | **0.94 (0.93-0.96)** |
|  | **135 to < 138** (n=611,947) | **0.91 (0.89-0.93)** | **1.04 (1.01-1.08)** | **1.04 (1.02-1.05)** |
| **Presence of Hyponatremia (<135 mEq/L), n=334,318** | **130 to < 135** (n=280,970) | **1.53 (1.50-1.57)** | **1.65 (1.59-1.70)** | **1.21 (1.19-1.23)** |
|  | **125 to < 130** (n=41,953) | **2.63 (2.52-2.74)** | **2.66 (2.51-2.82)** | **1.35 (1.31-1.39)** |
|  | **120 to < 125** (n=8,982) | **3.17 (2.93-3.44)** | **3.28 (2.95-3.66)** | **1.25 (1.18-1.33)** |
|  | **< 120** (n=2,413) | **4.72 (4.11-5.41)** | **3.79 (3.09-4.64)** | **1.40 (1.24-1.58)** |
