# Supplementary material for: Low serum sodium levels at hospital admission: Outcomes among 2.3 million hospitalized patients
Source: PLoS One. 2018 Mar 22;13(3):e0194379. doi: 10.1371/journal.pone.0194379 (PMC5864034; doi:10.1371/journal.pone.0194379)
Supplement: S3 Table — CI = confidence interval. aSerum sodium levels corrected by adding 1.6 mEq/L for each 100 mg/dL above 100 mg/dL of the concomitantly measured serum glucose levels. bThe adjusted relative ratios were derived from linear regression model adjusted for age, gender, race, and the selected comorbidities and reasons for hospitalization (p<0.001 for all). (DOCX) [file pone.0194379.s003.docx]

| **Serum [Na] levels^a^ at Hospital Admission (mEq/L), n= 2,284,912** | | **Length of Stay among those Discharged to Home (n=1,739,780)** |
| --- | --- | --- |
|  |  | **Adjusted^b^ Length of Stay - Ratio Relative to Reference (95% CI)** |
| **Absence of Hyponatremia (≥135 to ≤145 mEq/L), n=1,950,594** | **143 to ≤ 145** (n=134,979) | **1.03 (1.02 – 1.03)** |
|  | **140 to < 143** (n=602,058) | **1 (reference)** |
|  | **138 to < 140** (n=601,610) | **1.05 (1.05 – 1.06)** |
|  | **135 to < 138** (n=611,947) | **1.12 (1.12 – 1.13)** |
| **Presence of Hyponatremia (<135 mEq/L), n=334,318** | **130 to < 135** (n=280,970) | **1.18 (1.17 – 1.18)** |
|  | **125 to < 130** (n=41,953) | **1.21 (1.20 – 1.22)** |
|  | **120 to < 125** (n=8,982) | **1.16 (1.14 – 1.19)** |
|  | **< 120** (n=2,413) | **1.29 (1.24 – 1.35)** |
