# Supplementary material for: Low serum sodium levels at hospital admission: Outcomes among 2.3 million hospitalized patients
Source: PLoS One. 2018 Mar 22;13(3):e0194379. doi: 10.1371/journal.pone.0194379 (PMC5864034; doi:10.1371/journal.pone.0194379)
Supplement: S4 Table — aThe percentages and the relative risk ratios (RRR) of in-hospital mortality are for each age group with the respective [Na] category. bThe relative risk ratios are derived from a multinomial logistic regression model adjusted for age, gender, race, and the selected comorbidities and reasons for hospitalization. CI = Confidence interval. p<0.001 for all except: c p = 0.45. (DOCX) [file pone.0194379.s004.docx]

| **Final Cohort, n= 2,284,912** | **Serum Sodium Categories, mEq/L** | | | | | | | |
| --- | --- | --- | --- | --- | --- | --- | --- | --- |
| **Age groups** | **< 120** (n=2,413) | **120 to <125** (n=8,982) | **125 to < 130** (n=41,953) | **130 to < 135** (n=280,970) | **135 to < 138** (n=611,947) | **138 to < 140** (n=601,610) | **140 to < 143** (n=602,058) | **143 to ≤ 145** (n=134,979) |
| **Age 18 to <45 (n=461,511)** | **n=209** | **n=657** | **n=3,675** | **n=47,147** | **n=142,670** | **n=134,810** | **n=112,739** | **n=19,604** |
| Mortality, n (%) | 27 (12.9%)**^a^** | 43 (6.5%) | 208 (5.7%) | 622 (1.3%) | 794 (0.6%) | 591 (0.4%) | 758 (0.7%) | 431 (2.2%) |
| Adjusted RRR**^b^** (95% CI) | **15.3 (9.6-24.3)** | **6.7 (4.8-9.5)** | **5.6 (4.7-6.7)** | **1.6 (1.4-1.7)** | **0.76 (0.69-0.84)** | **0.64 (0.57-0.71)** | **1 (Reference)** | **3.3 (2.9-3.7)** |
| **Age 45 to <65 (n=740,035)** | **n=905** | **n=2,989** | **n=12,811** | **n=89,157** | **n=199,810** | **n=200,104** | **n=194,784** | **n=39,475** |
| Mortality, n (%) | 116 (12.8%) | 281 (9.4 %) | 965 (7.5%) | 3,256 (3.6%) | 3,402 (1.7%) | 2,717 (1.4%) | 3,135 (1.6%) | 1,517 (3.8%) |
| Adjusted RRR (95% CI) | **7.4 (5.9-9.2)** | **5.0 (4.3-5.7)** | **3.5 (3.3-3.8)** | **1.7 (1.7-1.8)** | **0.93 (0.88-0.97)** | **0.81 (0.77-0.85)** | **1 (Reference)** | **2.4 (2.2-2.5)** |
| **Age 65 to <75 (n=404,848)** | **n=433** | **n=1,719** | **n=8,468** | **n=52,649** | **n=104,535** | **n=103,702** | **n=108,698** | **n=24,644** |
| Mortality, n (%) | 36 (8.3%) | 148 (8.6%) | 612 (7.2%) | 2,546 (4.8%) | 3,009 (2.9%) | 2,449 (2.4%) | 2,949 (2.7%) | 1,313 (5.3%) |
| Adjusted RRR (95% CI) | **3.4 (2.3-4.9)** | **3.1 (2.6-3.8)** | **2.4 (2.2-2.7)** | **1.6 (1.5-1.7)** | **0.98 (0.93-1.03)^c^** | **0.83 (0.79-0.88)** | **1 (Reference)** | **2.1 (1.9-2.2)** |
| **Age ≥75 (n=678,518)** | **n=866** | **n=3,617** | **n=16,999** | **n=92,017** | **n=164,932** | **n=162,994** | **n=185,837** | **n=51,256** |
| Mortality, n (%) | 81 (9.3%) | 267 (7.4%) | 1,156 (6.8%) | 5,051 (5.5%) | 6,748 (4.1%) | 5,880 (3.6%) | 8,270 (4.4%) | 3,981 (7.8%) |
| Adjusted RRR (95% CI) | **3.0 (2.3-3.9)** | **2.0 (1.7-2.3)** | **1.7 (1.6-1.8)** | **1.2 (1.2-1.3)** | **0.89 (0.86-0.92)** | **0.78 (0.76-0.81)** | **1 (Reference)** | **2.0 (1.9-2.0)** |
| **Total mortality, n (%)** | **260 (10.8%)** | **739 (8.2%)** | **2,941 (7.0%)** | **11,475 (4.0%)** | **13,953 (2.3%)** | **11,637 (1.9%)** | **15,112 (2.5%)** | **7,242 (5.4%)** |
