# Supplementary material for: Low serum sodium levels at hospital admission: Outcomes among 2.3 million hospitalized patients
Source: PLoS One. 2018 Mar 22;13(3):e0194379. doi: 10.1371/journal.pone.0194379 (PMC5864034; doi:10.1371/journal.pone.0194379)
Supplement: S5 Table — RRR = Relative Risk Ratio. Age0 = Age (≥18 to <45); Age1 = Age (≥45 to <65); Age2 = Age (≥65 to <75); Age3 = Age ≥75. (DOCX) [file pone.0194379.s005.docx]

| **Age groups comparisons** | **<120 mEq/L** | | **120 to <125 mEq/L** | | **125 to<130 mEq/L** | | **130 to <135 mEq/L** | | **135 to <138 mEq/L** | | **138 to <140 mEq/L** | | **143 to ≤145 mEq/L** | |
| --- | --- | --- | --- | --- | --- | --- | --- | --- | --- | --- | --- | --- | --- | --- |
|  | **RRR (95% CI)** | **P value** | **RRR (95% CI)** | **P value** | **RRR (95% CI)** | **P value** | **RRR (95% CI)** | **P value** | **RRR (95% CI)** | **P value** | **RRR (95% CI)** | **P value** | **RRR (95% CI)** | **P value** |
| **Age0-Age1** | 2.07(1.24-3.46) | 0.005 | 1.34(0.93-1.95) | 0.12 | 1.58(1.31-1.91) | <0.001 | 0.89(0.79-1.01) | 0.07 | 0.82(0.73-0.91) | <0.001 | 0.79(0.70-0.89) | <0.001 | 1.38(1.20-1.58) | <0.001 |
| **Age1- Age2** | 2.19(1.43-3.37) | <0.001 | 1.58(1.25-2.00) | <0.001 | 1.46(1.29-1.66) | <0.001 | 1.11(1.03-1.20) | 0.005 | 0.95(0.88-1.02) | 0.19 | 0.97(0.90-1.05) | 0.45 | 1.14(1.04-1.26) | 0.007 |
| **Age 2-Age3** | 1.11(0.71-1.74) | 0.64 | 1.58(1.25-2.00) | <0.001 | 1.42(1.26-1.60) | <0.001 | 1.26(1.18-1.35) | <0.001 | 1.10(1.03-1.17) | 0.005 | 1.06(0.99-1.13) | 0.07 | 1.05(0.97-1.14) | 0.24 |
| **Age0-Age2** | 4.55(2.51-8.24) | <0.001 | 2.13(1.44-3.14) | <0.001 | 2.32(1.91-2.82) | <0.001 | 1.00(0.88-1.13) | 0.96 | 0.78(0.69-0.87) | <0.001 | 0.77(0.68-0.87) | <0.001 | 1.57(1.36-1.81) | <0.001 |
| **Age0-Age3** | 5.07(3.00-8.58 | <0.001 | 3.36(2.33-4.87) | <0.001 | 3.30(2.75-3.96) | <0.001 | 1.26(1.12-1.41) | <0.001 | 0.85(0.76-0.95) | 0.003 | 0.81(0.73-0.91) | <0.001 | 1.65(1.45-1.88) | <0.001 |
| **Age1-Age3** | 2.44(1.75-3.40) | <0.001 | 2.50(2.06-3.04) | <0.001 | 2.08(1.87-2.31) | <0.001 | 1.41(1.32-1.50) | <0.001 | 1.04(0.98-1.11) | 0.18 | 1.03(0.97-1.10) | 0.35 | 1.20(1.11-1.30) | <0.001 |
