# Supplementary figures and images for: Low serum sodium levels at hospital admission: Outcomes among 2.3 million hospitalized patients
Source: PLoS One. 2018 Mar 22;13(3):e0194379. doi: 10.1371/journal.pone.0194379 (PMC5864034; doi:10.1371/journal.pone.0194379)

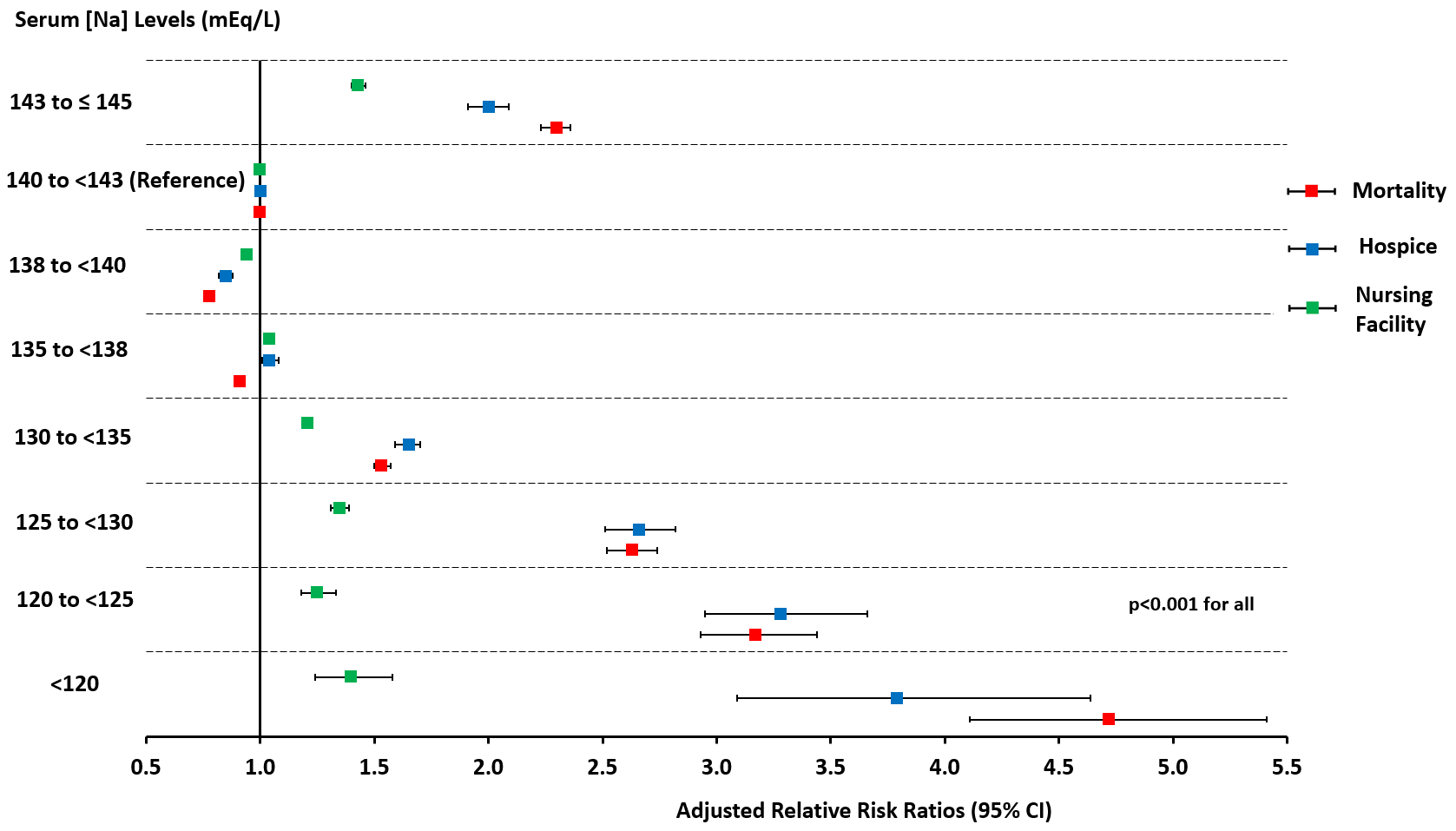

Supplement: S1 Fig — The relative risk ratios were derived from multinomial logistic regression models adjusted for age, gender, race, and Deyo-CCI. Discharge to home and serum sodium levels of (140 to <143 mEq/L) served as referent. Serum sodium levels were corrected by adding 1.6 mEq/L for each 100 mg/dL increase above 100 mg/dL of the concomitantly measured serum glucose levels. Error bars indicated 95% CI. CI = confidence interval; Deyo-CCI = Deyo Charlson Comorbidity Index. (DOCX) [file pone.0194379.s006.docx]

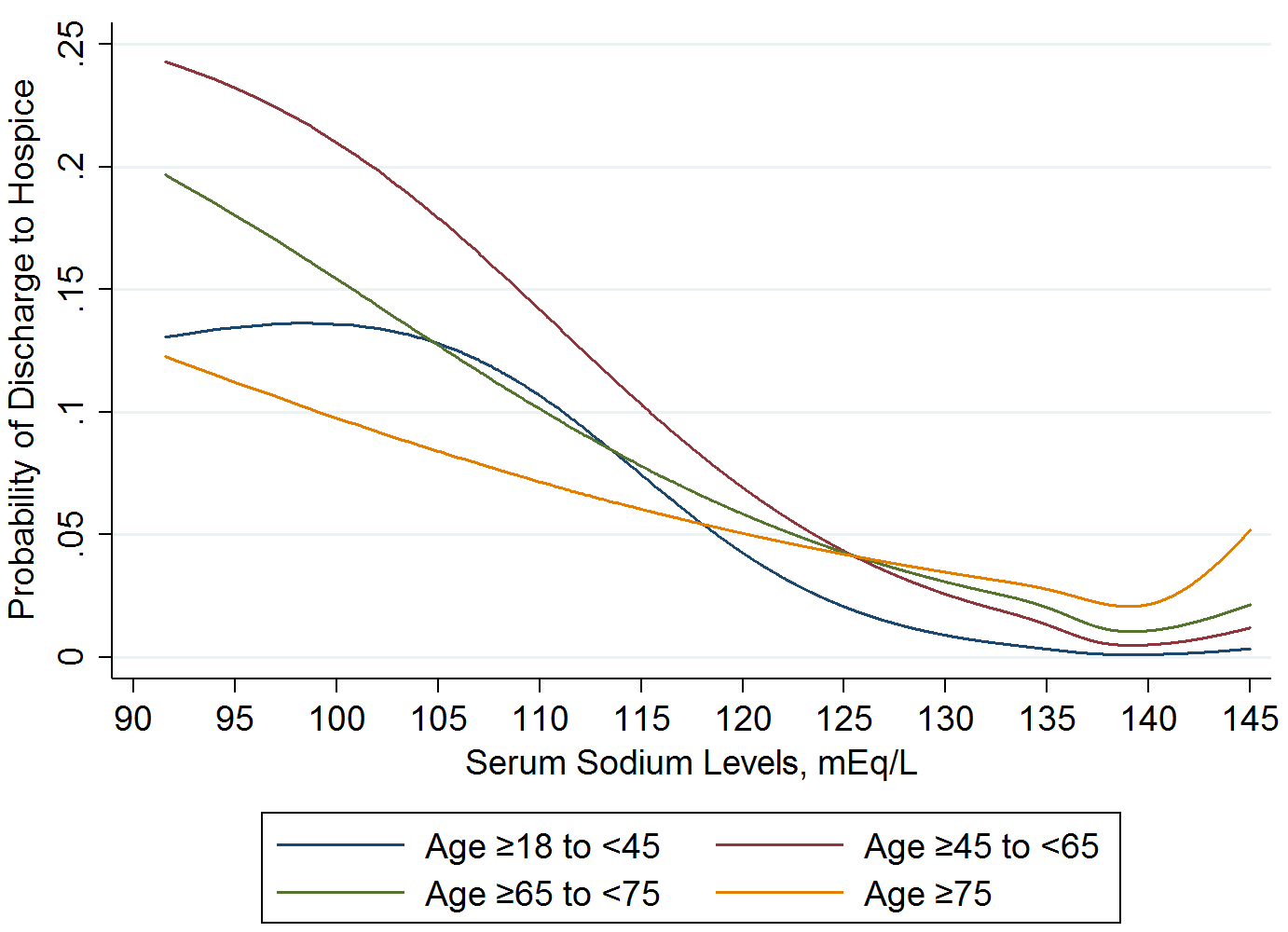

Supplement: S2 Fig — These estimated probabilities were derived from a multinomial logistic regression models stratified by age. (DOCX) [file pone.0194379.s007.docx]

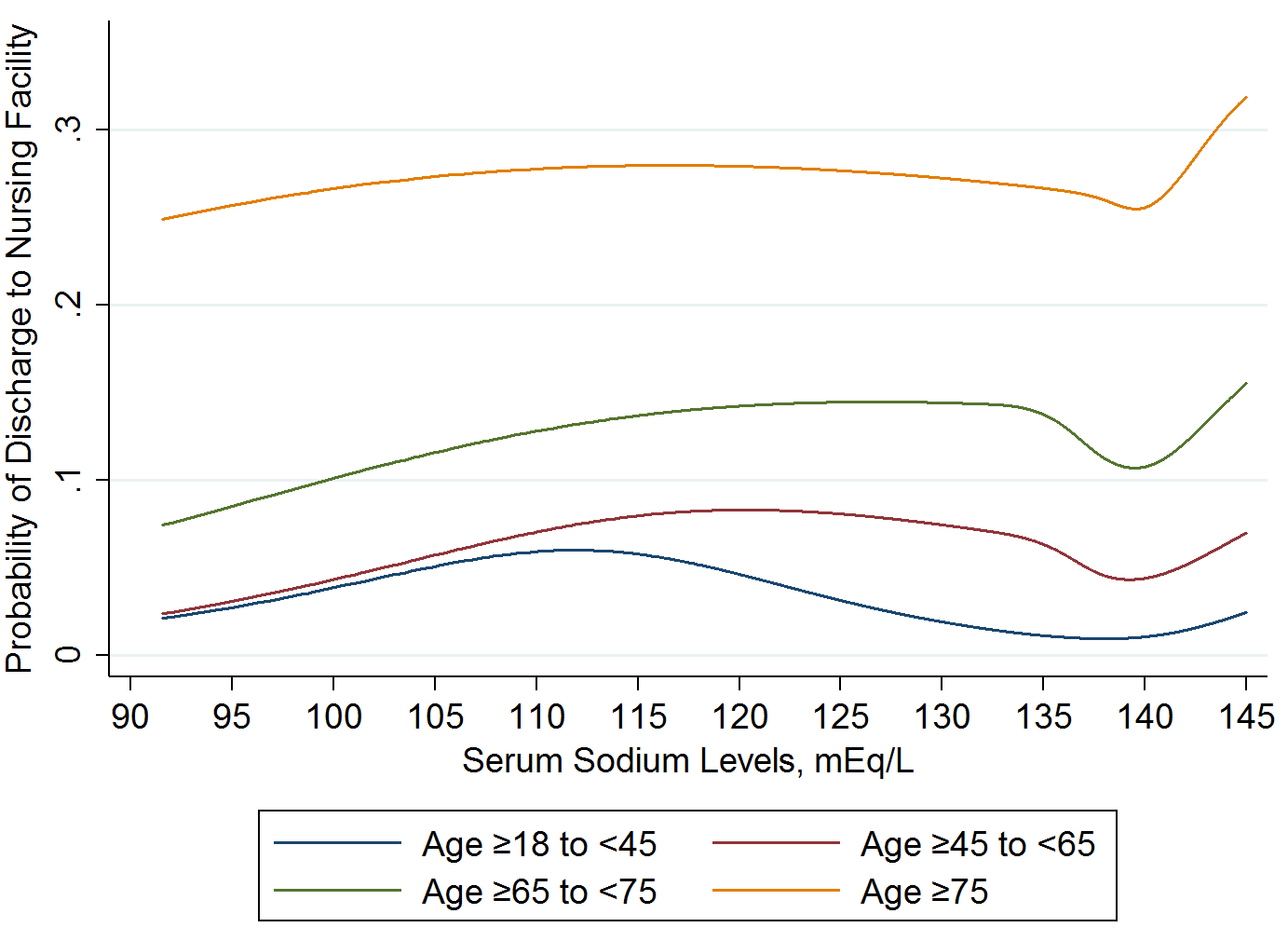

Supplement: S3 Fig — These estimated probabilities were derived from a multinomial logistic regression models stratified by age. (DOCX) [file pone.0194379.s008.docx]

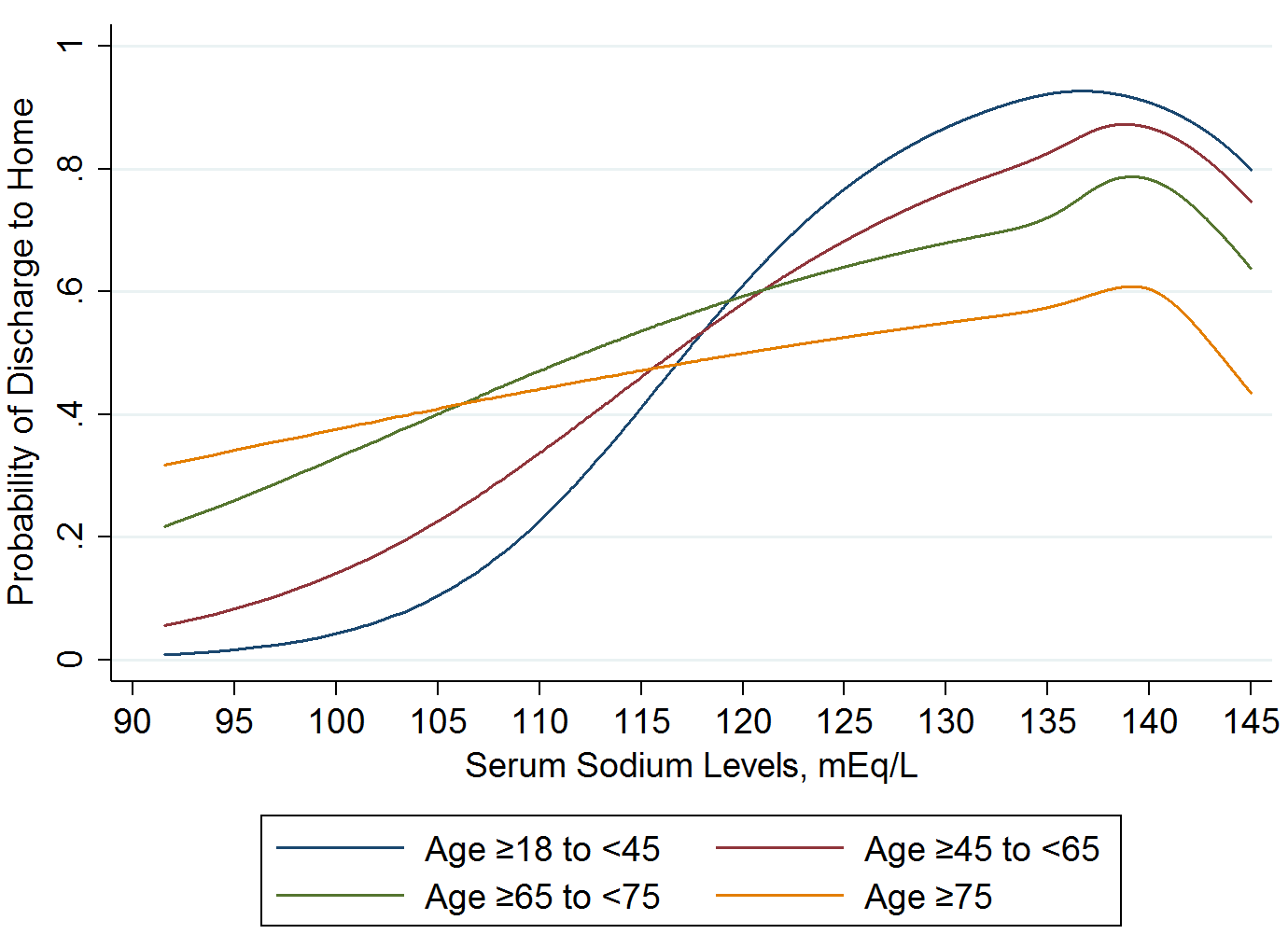

Supplement: S4 Fig — These estimated probabilities were derived from a multinomial logistic regression models stratified by age. (DOCX) [file pone.0194379.s009.docx]

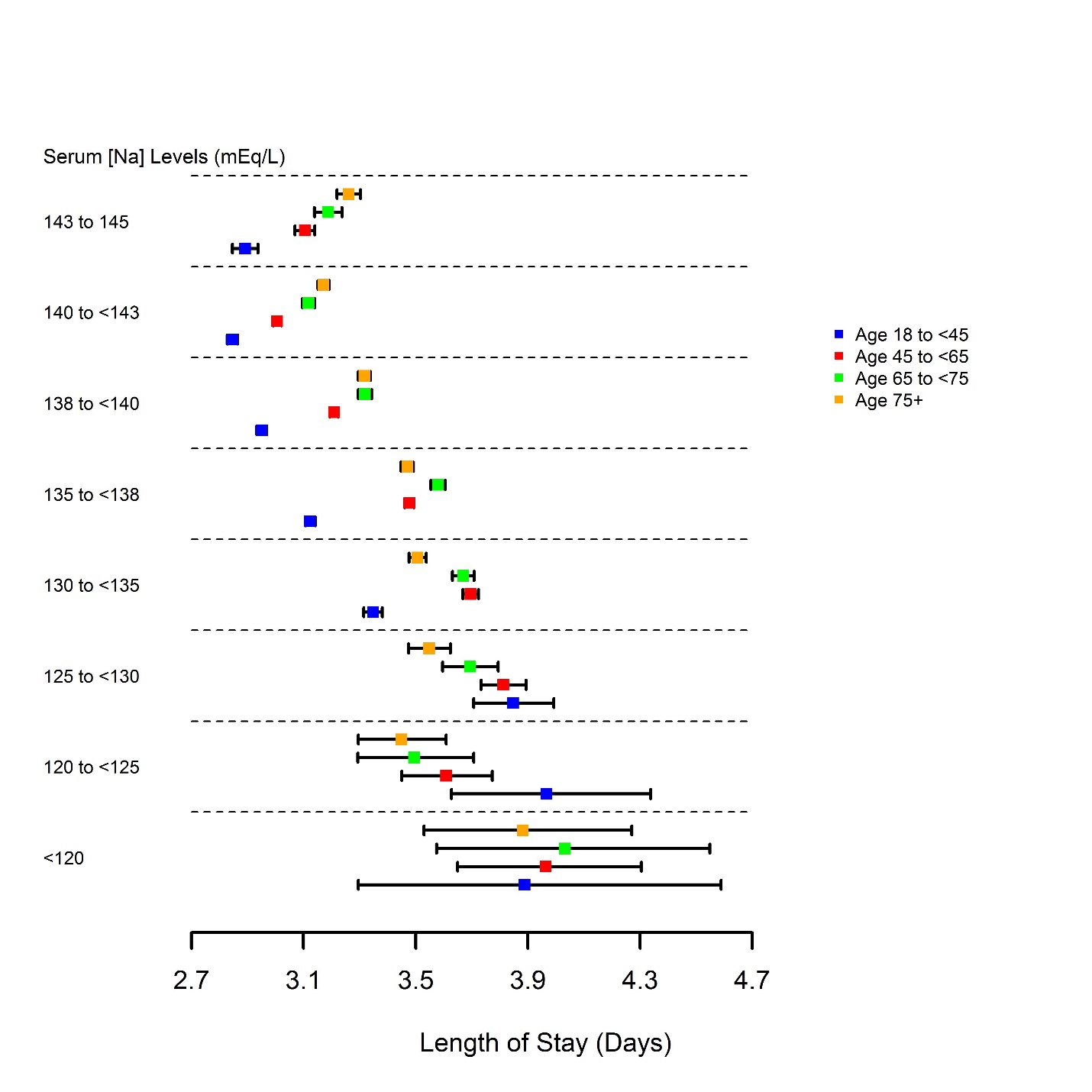

Supplement: S5 Fig — These estimated geometric means were derived from a linear regression model on the logarithmic-transformed number of days of hospitalization on [Na] levels with age categories included both as main effects, and in interactions with [Na] levels, while also adjusting for age, gender, race, and Deyo-CCI. (DOCX) [file pone.0194379.s010.docx]
